# Therapeutic targeting of PARP with immunotherapy in acute myeloid leukemia

**DOI:** 10.3389/fphar.2024.1421816

**Published:** 2024-08-08

**Authors:** Xing Bian, Wenli Liu, Kaijin Yang, Chuanbo Sun

**Affiliations:** ^1^ College of Biological and Pharmaceutical Engineering, West Anhui University, Lu’an, China; ^2^ Food and Drug Inspection Center, Lu’an, China; ^3^ Food and Drug Inspection Center, Huai’nan, China

**Keywords:** PARP, DNA repair, AML, immuotherapy, synergisctic effects

## Abstract

Targeting the poly (ADP-ribose) polymerase (PARP) protein has shown therapeutic efficacy in cancers with homologous recombination (HR) deficiency due to BRCA mutations. Only small fraction of acute myeloid leukemia (AML) cells carry BRCA mutations, hence the antitumor efficacy of PARP inhibitors (PARPi) against this malignancy is predicted to be limited; however, recent preclinical studies have demonstrated that PARPi monotherapy has modest efficacy in AML, while in combination with cytotoxic chemotherapy it has remarkable synergistic antitumor effects. Immunotherapy has revolutionized therapeutics in cancer treatment, and PARPi creates an ideal microenvironment for combination therapy with immunomodulatory agents by promoting tumor mutation burden. In this review, we summarize the role of PARP proteins in DNA damage response (DDR) pathways, and discuss recent preclinical studies using synthetic lethal modalities to treat AML. We also review the immunomodulatory effects of PARPi in AML preclinical models and propose future directions for therapy in AML, including combined targeting of the DDR and tumor immune microenvironment; such combination regimens will likely benefit patients with AML undergoing PARPi-mediated cancer therapy.

## Background

The poly (ADP-ribose) polymerase (PARP) protein superfamily comprises 17 members, which are encoded by different genes but share a common catalytic domain (Ame et al., 2004). PARP-mediated PARylation of PARP proteins themselves, or other DNA damage response (DDR) substrates, enhances DNA damage repair and promotes the survival of proliferating cells (Curtin and Szabo, 2020). The use of PARP inhibitors (PARPi) to treat *BRCA1/2*-mutant or homologous recombination (HR)-deficient tumors has been studied in various cancers, and is referred to as ‘synthetic lethality’ (Bryant et al., 2005; Farmer et al., 2005; Parvin et al., 2019).

PARP enzymes have important roles in cell biology processes, including post-transcription regulation, chromatin structure stabilization, metabolism, antiviral responses, telomere maintenance, cell cycle progression, and, most importantly, the DDR (Jubin et al., 2016). To date, eight PARP family members have been demonstrated to contribute to maintenance of genome stability through promoting DNA damage repair or cell cycle regulation (Slade, 2019). Besides being involved in single-strand break repair (SSBR), PARP proteins also have key roles in double-strand break (DSB) repair (Chen et al., 2018). In response to DNA damage, the PARP enzyme first localizes to damage sites *via* its N-terminal zinc finger domain (Langelier et al., 2011). The most important step in the repair process is the generation of PAR chains in multiple protein substrates, referred to as PARylation. Among PARP family members, PARP1 is the most abundant and responsible for generation of the majority of cellular PAR chains, while PARP2 accounts for only 5%–10% (Schreiber et al., 2006). The PARylation of multiple protein substrates leads to recruitment of DNA repair proteins to damage sites, where they mediate the repair cascade. Without appropriate PARP activity, single-strand breaks (SSBs) ultimately lead to formation of DSBs, which represent the most deleterious type of genome damage (Altmeyer et al., 2009; Bryant et al., 2009; Schiewer et al., 2012).

The PARP family members, PARP1, PARP2, and PARP3, are the most widely studied in mammalian cells, because of their involvement in DNA repair activity. These proteins act as DNA damage sensors, according to different types of DNA damage, and cooperate with other mediators to repair DNA damage through activating several DNA repair pathways, such as SSBR, HR, and non-homologous end joining (NHEJ) (Hartlerode and Scully, 2009; Pardo et al., 2009). In the following section, we review the function of PARP proteins, and particularly the PARP1 enzyme, in SSB and DSB repair. We also summarize recent studies using PARPi to treat acute myeloid leukemia (AML), where the majority of AML tumors harbor wild-type *BRCA1/2*.

### PARP enzymes in SSBR

**FIGURE 1:**
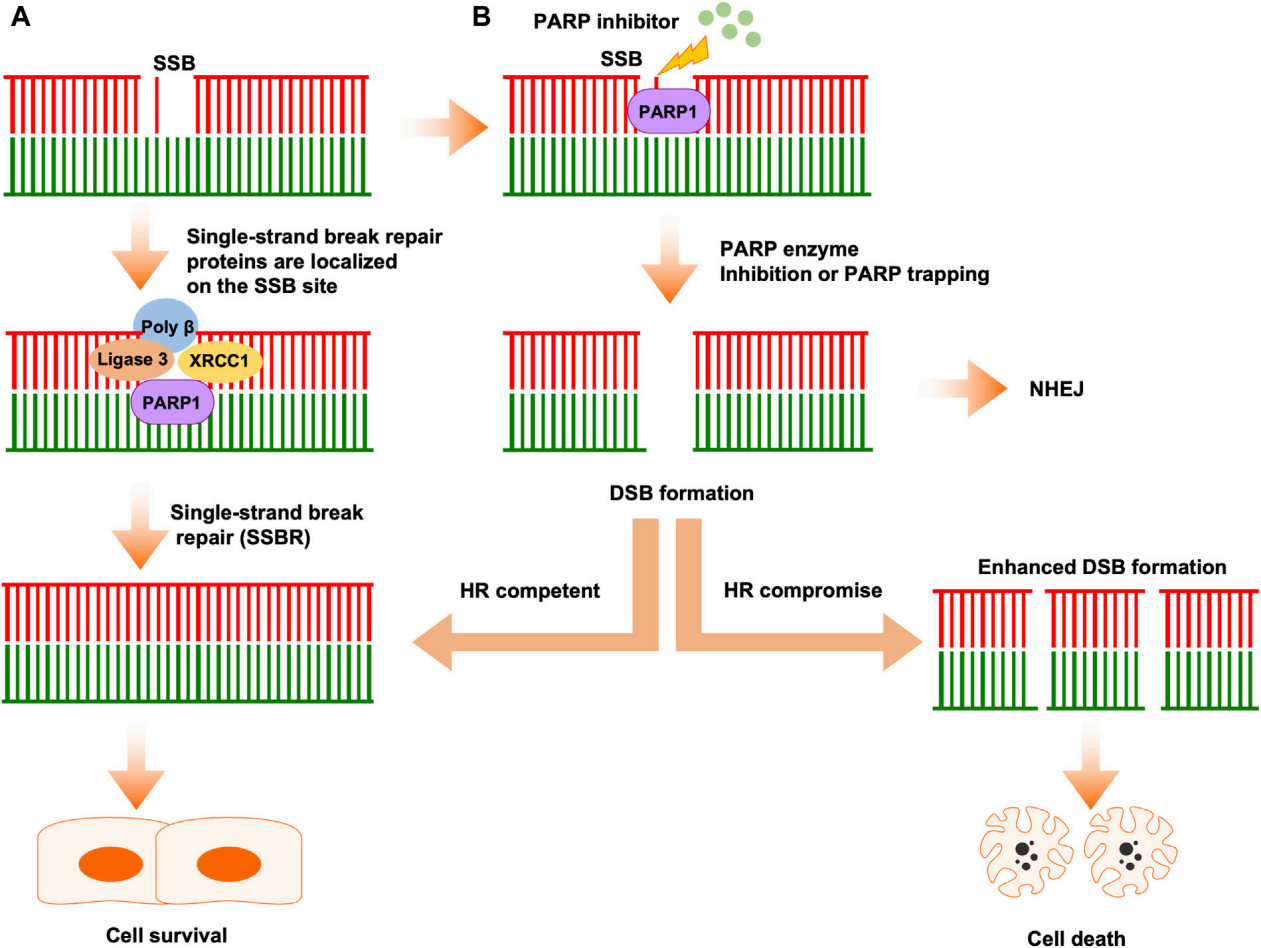
The classical mechanism of PARPi activity in cancer therapy. **(A)** Single-strand breaks are identified and repaired by PARP1. **(B)** PARPi induces double-strand break (DSB) formation by inhibiting PARP enzyme activity or PARP trapping. In HR-competent cells, DSBs can be repaired by HR, leading to cell survival. In HR-deficient cells, DSBs cannot be repaired correctly by error-prone NHEJ, leading accumulation of replication fork collapse and, ultimately, cell death.

In mammalian cells, there are thousands of DNA breaks arose per cell each day. In response to those DNA lesions, which include SSB and DSB, cells have evolved a series of DNA repair systems to maintain genome integrity. Endogenous factor-induced SSB can be repaired by PARP1-mediated base excision repair (BER) (Caldecott, 2008). In this process, PARP1 PARylates itself and a series of substrates to promote the accumulation of DNA repair factors at single-stranded DNA (ssDNA) sites to mediate repair progression (El-Khamisy et al., 2003; Ronson et al., 2018). In detail, upon PARylation, PARP1 collaborates with proteins, including DNA polymerase β, DNA ligase III, XRCC1, ALC1, and PNKP, to complete DNA break repair (Figure 1) (Abbotts and Wilson, 2017; Hanzlikova et al., 2017; Demin et al., 2021; Hewitt et al., 2021; Paes Dias et al., 2021).

### PARP enzyme in DSB repair

When SSBs are left unrepaired, or repaired incorrectly, they will convert into DSBs, which are the most dangerous type of lesion and threaten genome integrity, potentially leading to cancer development. Mammalian cells have involved two repair pathways which includes HR pathway and NHEJ pathway to repair the DSBs (Figure 1) (San Filippo et al., 2008; Hartlerode and Scully, 2009; Pardo et al., 2009). The phase of the cell cycle determines which pathway is used to repair the DNA lesions, NHEJ is active throughout the cell cycle, while HR only occurs in S or G2 phase (Huertas et al., 2008; Chapman et al., 2012; Orthwein et al., 2014). In response to DSB, PARP1 is among the proteins that respond earliest to DSBs and, once PARP1 is activated by DSB, it recruits initial mediators to DSB sites.

NHEJ pathways can be divided into classical and alternative types. Classical NHEJ (cNHEJ) always repairs breaks occurring in the G0/G1 phase, during which PARP1 PARylates DNA-PKCs and PARylation of PARP1 itself recruits the KU70-KU80 complex, which promotes DNA ligase IV and the XRCC4/XRCC4-like factors (XLF) complex to DNA ends, to mediate their ligation (Lieber, 2010; Chang et al., 2017; Han et al., 2019). Notably, before ligation, DNA ends require processing by the nucleases, Artemis and APLF (Davis and Chen, 2013). Compared with cNHEJ, alternative NHEJ (aNHEJ) is active in the S and G2 phases of the cell cycle (Wyatt et al., 2016; Yu et al., 2020). The first step of this pathway is initiated by PARP1-mediated localization of the MRN-CtIP complex on the DNA ends, then CtIP acts with MRN to mediate the excision of DNA ends, to expose ssDNA microhomology sequences (Xie et al., 2009; Anand et al., 2016). Following this step, PARP1 combines with MRN and Pol θ to promote alignment of DNA single strands through microhomology sequences (Kent et al., 2015). For DNA regions without 3′homology sequences, ends are digested by XRCC1 and XPF (Okano et al., 2005). Overall, PARP1 is indispensable, and importantly interacts with various other factors, to complete both the cNHEJ and aNHEJ repair processes.

Compared with NHEJ repair, in which DNA ends are directly ligated and always recognized in an error-prone manner, HR is highly accurate and uses the sister chromatid as template to complete DNA repair (Juhasz et al., 2018; Ranjha et al., 2018). This characteristic restricts HR to occurring only in S and G2 phase, when sister chromatids are available (Liu and Huang, 2014). The first step of HR is initiation of DNA end resection, which is mediated by the MRN complex and BRCA1 (Bunting et al., 2010; Cruz-Garcia et al., 2014; Zhao et al., 2020). The role of PARP1 in this process is to recruit and activate the MRN complex and BRCA1 to the DSB ends (Li and Yu, 2013). However, there have also been reports that BRCA1 localization to DSB ends is independent of PARP1 (Wu et al., 2009). DNA end resection leads to subsequent generation of ssDNA, which is first bound by RPA, followed by recruitment and loading of RAD51 on to the ssDNA to complete the repair process (Chen et al., 2013; Prakash et al., 2015). DNA end resection is a fundamental step in HR and its efficiency is determined by PARP1, which PARylates various substrates, including CtIP, the MRN complex, and BRCA1 (Moison et al., 2021; Luedeman et al., 2022). Notably, BRCA1 and 53BP1 play opposing roles in determining which DSB repair pathway (HR or cNHEJ) occurs (Escribano-Diaz et al., 2013).

## Rationale for targeting PARP in AML

Sequence data demonstrate that PARP family genes alteration is rare in patients with AML; however, mutations and copy number alterations of DDR genes, such as, *ATM, ATR, CHEK1*, *CHEK2, RAD51*, and *PALB2*, have been detected, leading to HR pathway dysregulation in patients with AML (cBioPortal for Cancer Genomics. http://www.cbioportal.org/.). *PALB2* deletion predicts HR defects and confers cancer cell sensitivity to PARPi (Grellety et al., 2020; Carreira et al., 2021; Dillon et al., 2022). Further, there is mounting evidence that PARP family members have key roles in regulating gene transcription, promoting protein stability, and modulating chromosome structure (Wacker et al., 2007; Ji and Tulin, 2010; Krishnakumar and Kraus, 2010). Dysregulation of gene transcription or chromosomes can result in errors in the DDR. Collectively, this led to the realization that targeting PARP may provide an ideal strategy for AML patients with known genetic background such as different karyotypic aberrations (Tyner et al., 2018). In the following sections, we summarize preclinical and clinical studies of different PARPi for treatment of AML (Table 1).

**TABLE 1:** Ongoing clinical trials of the use of PARP inhibitors in AML.

Inhibitor	Combination with	Phase	Status	NCT number
Talazoparib	Topotecan/gemcitabine	Phase I	Rrecruiting	NCT05101551
Talazoparib	Decitabine	Phase I and II	Completed	NCT02878785
Olaparib	Monotherapy	Phase II	Active	NCT03953898
Veliparib	Temozolomide	Phase I	Active	NCT01139970
Talazoparib	Monotherapy	Phase I	Completed	NCT01399840

Immunotherapy for AML.

### Olaparib

**FIGURE 2:**
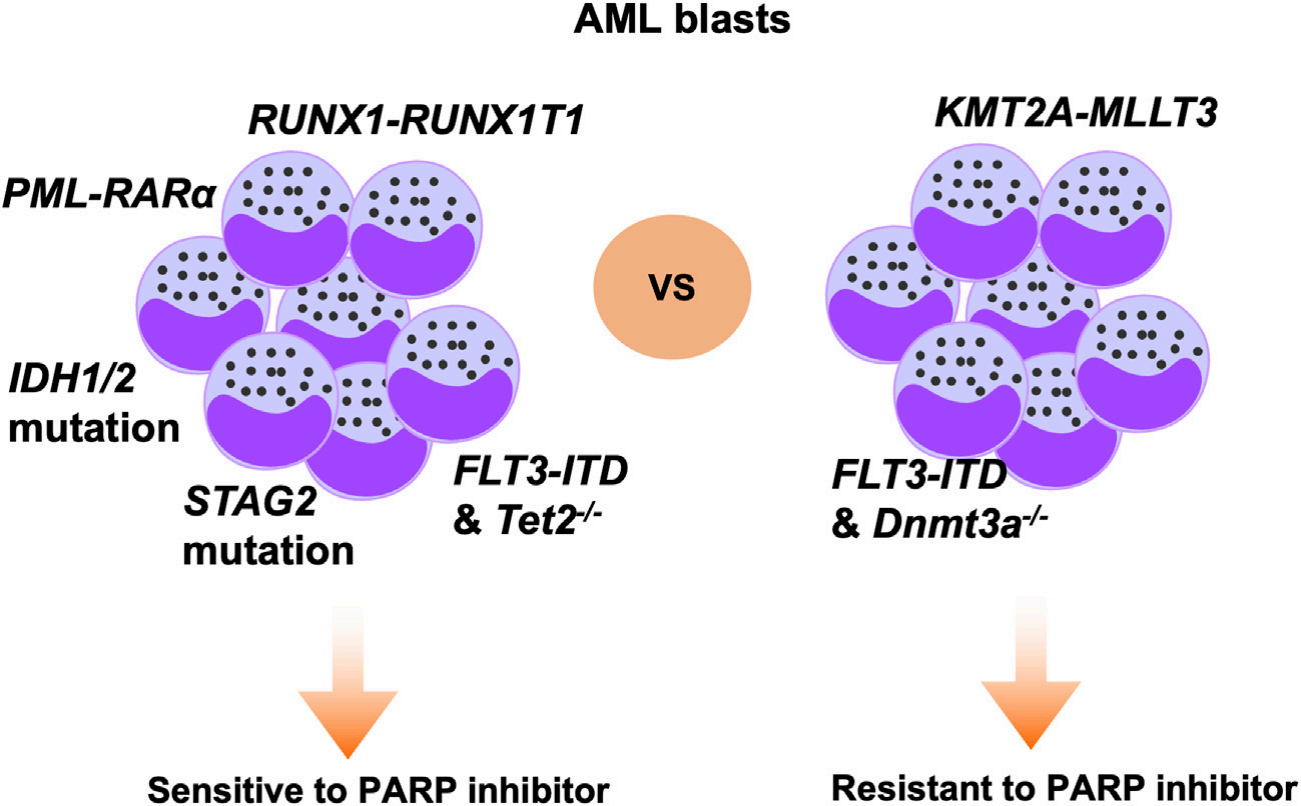
Specific genetic alterations that modify therapeutic sensitivity to PARPi in AML. Based on previous studies, AML cells carrying mutations of, or deficient for, *RUNX1-RUNX1T1*, *PML-RARα*, *IDH1/2*, *STAG2*, and *FLT3-ITD/Tet2* ^*−/−*^ genes are sensitive to PARP inhibition. In contrast, AML cells carrying *KMT2A-MLLT3* or *FLT3-ITD/Dnmt3a* ^*−/−*^ are not responsive to PARP inhibition.

As we known, SSB frequently occurs in proliferating cells, and efficient repair of SSB is dependent on PARP activity. The mechanism underlying PARPi activity can be divided into two aspects: inhibition of PARP enzyme activity and tapping PARP itself on the damage sites. Olaparib inhibits four members of the PARP family, PARP1 to PARP4, and is the PARPi that has been most extensively studied in the context of AML (Casorelli et al., 2006). In a previous study, patient-derived AML cells carrying AML1-ETO or PML-RARα mutations were demonstrated to be sensitive to olaparib. In contrast, MLL-AF9 or E2A-PBX subtype AML cells were unresponsive to olaparib. Mechanistic analysis showed that AML1-ETO or PML-RARα fusion oncogenes can inhibit HR activity through suppressing several HR-associated genes in AML cells, thus leading to olaparib sensitivity (Figure 2) (Esposito et al., 2015). In another study, olaparib inhibited XRCC1 loading onto DNA damage sites and prevented BER repair, while combination treatment with olaparib and decitabine induced synthetic lethality effects in AML cells (Orta et al., 2014). WEE1 inhibitor has been shown to sensitize cancer cells to cytotoxic agents, and WEE1 inhibition can reduce HR activity by directly constraining BRCA2 (Kausar et al., 2015). Garcia et al. showed that the WEE1 inhibitor, AZD1775, combined with olaparib, induced synergistic antitumor effects in an AML model (Garcia et al., 2017). Mechanistic analysis further demonstrated that AZD1775 suppressed HR and enhanced DNA damage, thus sensitizing AML cells to PARPi. Mutations of FLT3 have been detected in up to 23% of patients with AML and confer a poor prognosis. In a recent study, the FLT3 kinase inhibitor, AC220, was used to treat FLT3-positive AML cells, and was found to suppress expression of a subset of DNA repair proteins, thus causing synthetic lethal effects when administered with olaparib. Combined use of AC220 and olaparib eliminated FLT3-positive quiescent and proliferating leukemia stem cells, as well as reducing leukemia initiating cells (Maifrede et al., 2018). NF-κB is reported to mediate resistance of AML cells to DNA damage agents (Faraoni et al., 2018). Ding et al. showed that simultaneous targeting of NF-κB and PARP with olaparib resulted in substantial cell killing (Li et al., 2019). *IDH1/2* mutation is reported to occur in 20% of patients with AML and inhibits DNA damage repair genes, conferring sensitivity of cancer cells to PARPi (Sulkowski et al., 2017; Fritz et al., 2021). In a recent paper, the authors showed that *IDH1/2*-mutated AML cells were sensitive to olaparib or talazoparib, and the study further supported the clinical trial of olaparib monotherapy or combined with daunorubicin in AML patients with *IDH1/2*-mutation (Figure 2) (Molenaar et al., 2018). Furthermore, olaparib is reported to be effective against *IDH1/2*-mutated AML or MDS in patient-derived xenograft models, but not in corresponding wild-type AML/MDS models (Gbyli et al., 2022). Together, these studies support the translation of PARPi for application in patients with AML with *IDH1/2*-mutation. KDM6A loss-of-function mutation was reported to be associated with conventional chemotherapy response in patients with AML, and olaparib treatment has demonstrated antitumor efficacy in AML with KDM6A mutation. Co-targeting PARP and BCL2 using olaparib in AML showed superior therapeutic effects (Boila et al., 2023). Further, vitamin C can inhibit AML progression through enhancing TET2 activity; however, single agent modality treatment was not curative in this disease. Nevertheless, a recent study demonstrated that treatment with vitamin C combined with olaparib elicited strong synergistic effects in blocking AML self-renewal in murine and human AML models (Brabson et al., 2023). Together, these results demonstrate the promising therapeutic potential of olaparib in AML, either as single agent or combination with other inhibitors.

### Rucaparib and niraparib

Rucaparib and niraparib are small molecular inhibitors administered orally. Rucaparib targets PARP1 to PARP4 (Syed, 2017), while niraparib is designed to target PARP1 and PARP2 for cancer therapy (Shen et al., 2015). Few studies have tested the efficacy of rucaparib or niraparib in AML. One investigation using rucaparib with the cytotoxic agent, 5-FU, to treat acute leukemias detected strong synergistic antitumor efficacy against AML (Falzacappa et al., 2015). Comparably, a triple combination of niraparib, decitabine, and HDACi synergistically induced DNA damage and promoted cell death in AML cell lines, the effects of this triple combination on primary leukemic cells were also confirmed (Valdez et al., 2018).

### Talazoparib

Talazoparib has the most potent PARP trapping activity compared with other PARP inhibitors (Murai et al., 2012). There are several studies that test the efficacy of talazoparib against AML. Talazoparib or APE1 inhibitor were demonstrated to induce critical antileukemic effects against selected primary CD34^+^ AML samples, and further experiments demonstrated that low dose talazoparib and APE1 inhibitor treatment enhanced the efficacy of decitabine against AML (Kohl et al., 2019). The cohesin complex plays an important role in DNA chromosome maintenance and transcription regulation (Jeppsson et al., 2014). Recurrent somatic alteration of the cohesin complex is frequent in AML, and cohesin-mutant cells are reported to be highly dependent on DNA damage repair and replication networks; hence, AML cells with cohesin complex mutations are sensitive to talazoparib (Tothova et al., 2021). PARP1 expression levels are correlated with prognosis in patients with cancer, and high PARP1 expression predicts poor survival of patients with AML, while combined treatment using talazoparib and NL101 resulted in strong synergistic effects against AML (Li et al., 2018). Mechanistic analysis showed enhanced cell apoptosis, G2 cell cycle arrest, and DNA damage in response to talazoparib and NL101 combination therapy. Further, DNA methyltransferase inhibitors substantially enhanced the efficacy of talazoparib against AML, both *in vitro* and *in vivo* (Muvarak et al., 2016). Moreover, in a recent clinical trial, the same research group conducted a dose escalating study of DNMTi, decitabine, combined with talazoparib for treatment of patients with AML who were previously treated or not treated with decitabine. The results indicated that, combined of decitabine with talazoparib is well-tolerated and that pharmacodynamic effects can be expected in responsive patients (Baer et al., 2022). BACT1, a key enzyme in branched-chain amino acid metabolism, has important roles in cancer progression; however, the oncogenic role of BACT1 in AML has not been fully elucidated. A recent study demonstrated that BACT1 can decrease DDR activity and sensitize AML cells to talazoparib, both *in vitro* and *in vivo* (Pan et al., 2024).

## Immunotherapy for AML

Over the past 4 decades, the standard treatment option for patients with AML is combination of chemotherapy. Fewer than one-third of patients with AML are responsive to core chemotherapy, meaning that most are unresponsive, and highlighting the need to identify new therapeutic approaches to satisfactorily treat more patients with this condition (Estey, 2014). Hematopoietic stem cell transplantation (alloHSCT) represents the most promising strategy for curing patients with AML; however, very few patients are eligible for this approach and, after alloHSCT, most develop tumor relapse, leading to particularly poor clinical outcomes (Christopher et al., 2018; Hansrivijit et al., 2019). A key reason underlying AML relapse is tumor cell escape from immune cell surveillance or attack (Zeiser and Vago, 2019; Tettamanti et al., 2022). In this section, we summarize recently developed novel strategies, particularly involving immune checkpoint blockade, for AML treatment, and discuss the obstacles to identifying targets for AML immunotherapy.

## Mechanisms of immune evasion in AML

**FIGURE 3:**
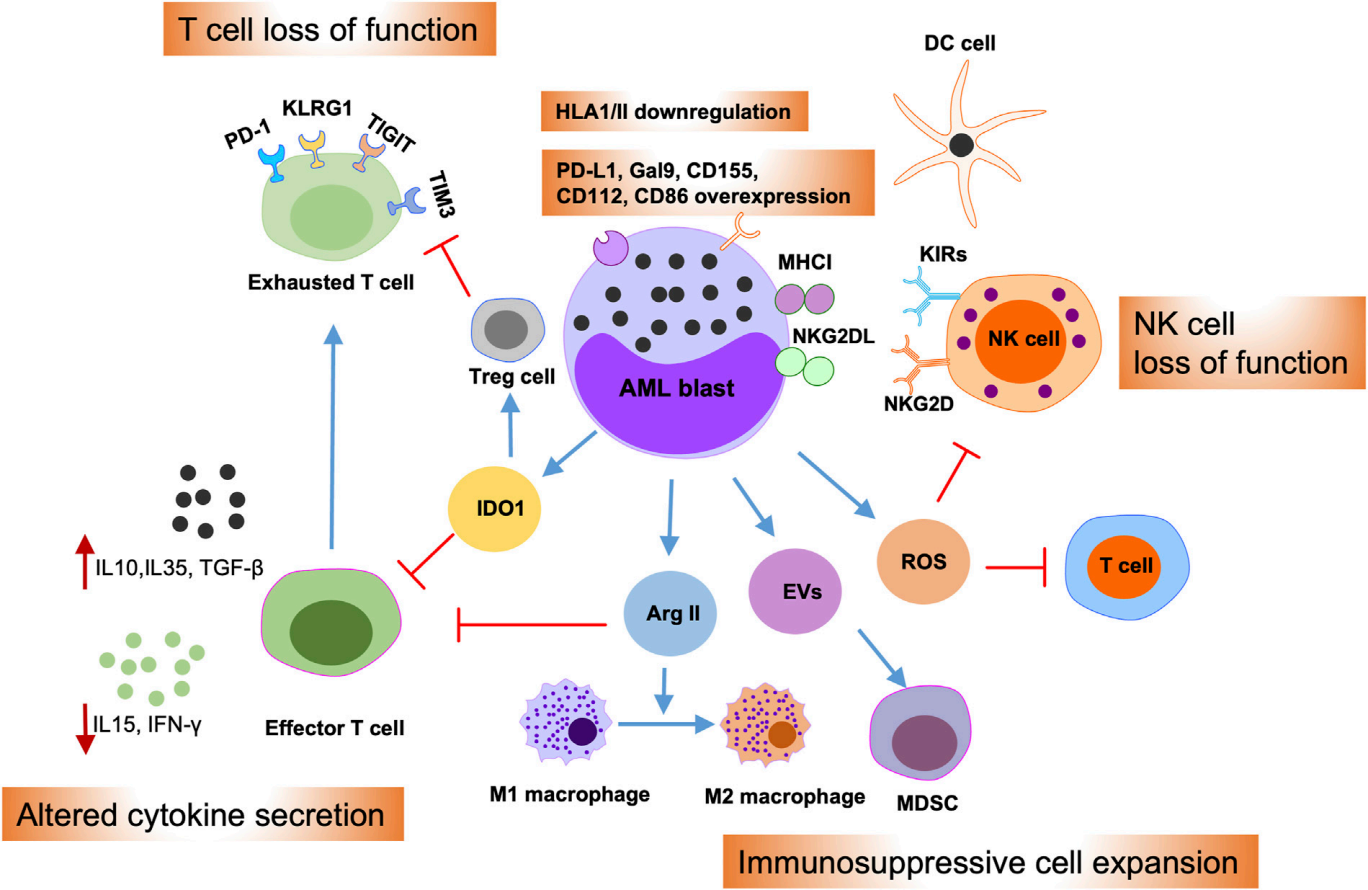
Immune evasion mechanisms of AML blasts. Schematic illustration summarizing intrinsic and extrinsic AML immune evasion mechanisms. AML blasts can impede T and NK cell effector functions by overexpressing inhibitory T cell ligands, such as PD-L1, Gal9, CD155, CD122, and CD86, or by overexpressing the NKG2D ligand, NKG2DL. Meanwhile, AML blasts can reduce the expression of antigen presentation molecules; thereby suppressing their presentation to dendritic cells (DCs). Furthermore, AML blasts can alter cytokine secretion in the microenvironment and release of reactive oxygen species (ROS), indoleamine 2,3-dioxygenase-1 (IDO1), arginase II (Arg II), and extracellular vesicles (EVs), into the bone marrow (BM) niche. This, in turn, can promote T cell exhaustion and apoptosis, drive the expansion of myeloid-derived suppressor cells (MDSCs) and regulatory T cells (Tregs), and induce switch of macrophages from M1 to the tumor-promoting M2 phenotype.

Multiple mechanisms are implicated in regulating AML immune evasion, including leukemia-intrinsic and -extrinsic evasion processes (Figure 3) (Vago and Gojo, 2020; Tettamanti et al., 2022). The first is that AML blasts can inhibit the expression of antigen presentation molecules and overexpress immune inhibitory molecules, such as PD-L1 and Cal-9 (Kikushige et al., 2010; Kikushige and Miyamoto, 2015; Taghiloo and Asgarian-Omran, 2021). Further, the bone marrow niche can release reactive oxygen species, indoleamine 2,3-dioxygenase 1, TGF-β, arginase, and extracellular vesicles in the AML microenvironment, which can suppress the cytotoxic effects of T and NK cells, as well as promoting regulatory T cell and myeloid-derived suppressor cell (MDSC) activity (Curti et al., 2009; Aurelius et al., 2012; Lu and Gabrilovich, 2012; Sun et al., 2015; Pyzer et al., 2017; Ding et al., 2018; Yang et al., 2020). Moreover, the AML microenvironment can promote M1-type macrophage conversion into M2-type macrophages, which promote cancer development (Al-Matary et al., 2016). Together, these intrinsic and extrinsic mechanisms cooperate to induce tumor cell immune evasion, thereby mediating therapy resistance.

## Immune checkpoint blockade inhibitors in AML

The efficacies of multiple immune checkpoint inhibitors in the treatment of AML have been studied (Table 2). An early investigation explored the effect of ipilimumab against hematologic malignancies, and found that a dose of 10 mg/kg induced promising responses in 5/12 patients with AML, among which 3 patients had responses lasting more than 1 year (Davids et al., 2016). The combined effects of ipilimumab combined with decitabine are now being explored in the clinic in both pre- and post-alloHSCT patients, in comparison with those of monotherapy. Hypomethylating agents (HMAs) have been shown to affect the immune system, and a combination of HMA with PD-1/PD-L1 inhibition has been evaluated in AML in several studies, with a subset of patients found to respond to the combination regimen (Daver et al., 2019). Furthermore, a triple combination of azacytidine, nivolumab, and ipilimumab led to improved outcomes, but with more frequent immune-related side effects (NCT02397720). Pembrolizumab, another PD-1 inhibitor, was evaluated for use in patients with AML in combination with decitabine or azacytidine, and the results were similar to those of combination therapy with azacitidine and nivolumab (Goswami et al., 2022) (NCT02845297). Further, targeting the PD-1/PD-L1 interaction using anti-PD-1 or -PD-L1 antibodies showed limited effects in patients with AML, while the combination of PD-1 or PD-L1 inhibitors and chemotherapy enhanced treatment efficacy in patients newly-diagnosed with AML.

**TABLE 2:** Clinical studies of the use of ICIs as monotherapy or combination therapy for AML.

Inhibitor	Combination with	Phase	Status	NCT number
Camrelizumab	Decitabine	Phase II	Unknown	NCT04353497
Nivolumab	Monotherapy	Phase II	Active	NCT02275533
Atezolizumab	Guadecitabine	Phase I/II	Active	NCT03935361
Atezolizumab	Hu5F9-G4	Phase I	Terminated	NCT03922477
Atezolizumab	Guadecitabine	Phase I	Completed	NCT02892318
Nivolumab	Decitabine/Venetoclax	Phase I	Active	NCT04277442
Atezolizumab	BL8040	Phase I/II	Terminated	NCT03154827
Nivolumab/Relatlimab	Azacitidine	Phase II	Recruiting	NCT04913922
Pembrolizumab	Monotherapy	Phase II	Completed	NCT02708641
Pembrolizumab	Azacitadine	Phase II	Completed	NCT02845297
Pembrolizumab	Decitabine	Phase I/II	Completed	NCT02996474
Nivolumab	Cytarabine	Phase II	Terminated	NCT03381118
Pembrolizumab	Azacitidine	Phase II	Recruiting	NCT03769532
Pembrolizumab	Fludarabine/Melphalan	Phase II	Completed	NCT02771197
Pembrolizumab	Azacitidine/Venetoclax	Phase II	Active	NCT04284787
Pembrolizumab	Cytarabine/idarubicin	Phase II	Recruiting	NCT04214249
Nivolumab/Azacitidine	ipilimumab	Phase II	Completed	NCT02397720
Nivolumab	azacitidine	Phase II/III	Active	NCT03092674
Pembrolizumab	cytarabine	Phase II	Active	NCT02768792
Nivolumab	Idarubicin/cytarabine	Phase I/II	Completed	NCT02464657
Pembrolizumab	Venetoclax/Decitabine	Phase I	Recruiting	NCT03969446
Pembrolizuma	AMG330	Phase I	Terminated	NCT04478695
Atezolizumab	gilteritinib	Phase I/II	Completed	NCT03730012
Ipilimumab	Decitabine	Phase I	Active	NCT02890329
Nivolumab/Ipilimumab	Monotherapy	Phase I	Active	NCT03600155
Nivolumab	cyclophosphamide	Phase II	Completed	NCT03417154
Ipilimumab	Monotherapy	Phase I	Active	NCT03912064
Ipilimumab	Monotherapy	Phase I	Completed	NCT01757639
Nivolumab	5-azacytidine	Phase I/II	Active	NCT03825367

## Immunomodulatory effects of PARP inhibitor in AML

**FIGURE 4:**
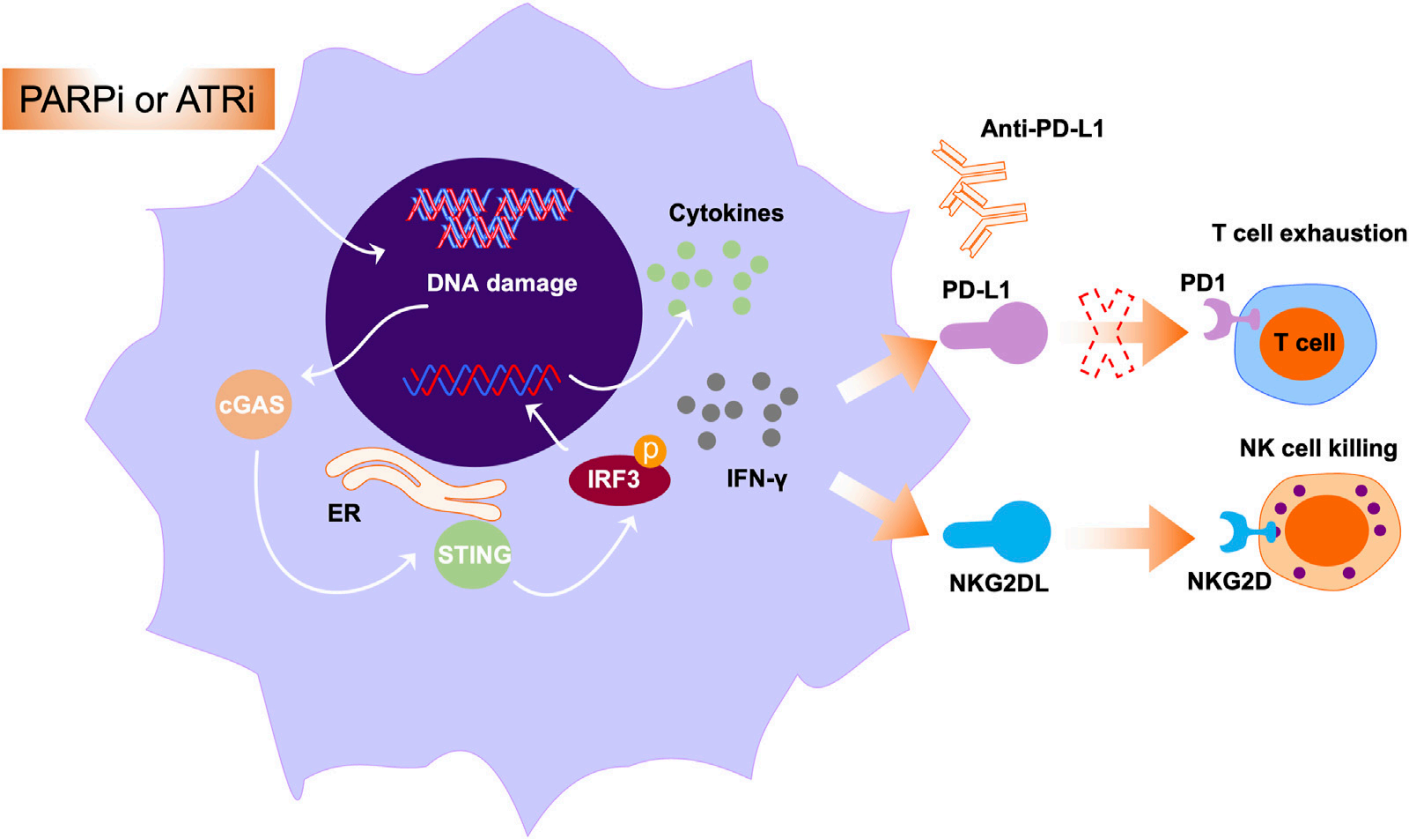
The roles PARPi or ATRi in immune modulation. Multiple studies have exploited the immunogenic properties of anticancer drugs to enhance tumor immunogenicity. The represented approach is through activating cytosolic immunity pathways. In this pathway, PARPi or ATRi activate cGAS-STING signals and mediate the secretion of IFN-γ and other cytokines. Further, IFN-γ secretion can induce NKG2DL expression, leading to interaction of NKG2DL with NKG2D and subsequently augmenting NK cell-mediated cell killing.

Conventionally, PARPi are considered to exert their functions through enzyme inhibition and PARP trapping. In addition to a direct effect on the DDR, there are increasing reports that PARP inhibition can modulate immune responses in the tumor microenvironment (Pham et al., 2021; Kornepati et al., 2022). PARP inhibition can enhance innate immunity through various mechanisms, leading to the development of combination treatments with PARPi and immunotherapy for the treatment of cancers, including solid tumors and hematologic malignancies (Ding et al., 2018; Kim et al., 2020). Dysregulation of the DDR occurs through various mechanisms, including enhancement of tumor mutational burden (TMB) by PARP inhibition, leading to generation of neoantigens, which can promote the cytotoxic effects of T cells (Dall’Olio et al., 2022). However, recent studies have demonstrated that PARP inhibition activates stimulators of inhibitory genes, further enhancing PD-L1 expression on the surface of tumor cells (Figure 4) (Sen et al., 2019). Collectively, PARP inhibition by enhanced tumor immunogenicity creates an ideal microenvironment for combination treatment of cancer using PARPi and immune checkpoint inhibitors.

In hematologic malignancy, the combination of PARP inhibitor with immune checkpoint inhibitors warrants further exploration. In a previous study, genotoxic stress such as PARP inhibition induced expression of NKG2D ligands in AML cells, and NKG2D ligands bind to NKG2D receptors in immune cells, including NK and cytotoxic T cells (Gasser et al., 2005). Binding of NKG2D ligands to the NKG2D receptor on the surface of NK cells can exert their cytotoxic effects, leading to NK cell-mediated cell killing (Figure 4). Paczulla et al. showed that PARP1 enzyme can repress expression of NKG2D ligands on the surface of AML cells. Therefore, treatment using PARPi, followed by transfer of NK cells, can substantially suppress leukemogenesis in AML patient-derived xenograft models (Paczulla et al., 2019). Moreover, PARPi can sensitize AML cells to TRAIL (a key NK cell effector molecule) through activating Fas and DR5 (Meng et al., 2014). Taken together, these findings open new avenues for treatment of AML using PARPi in combination with immunomodulatory agents.

## Conclusion and future directions

Patients with AML often achieve tumor remission after standard therapy, but invariably die of relapse via various mechanisms, such as the presence of leukemic stem cells (Stavropoulou et al., 2016). Preclinical studies are increasingly identifying and investigating novel therapeutics with potential to eradicate bulk AML cells and AML stem cells. PARPi, which are widely used to treat breast and ovarian cancers carrying BRCA mutations, are now emerging as novel therapeutics for acute leukemia with selected genetic backgrounds; for example, they are effective against *IDH1/2*- or *AML1-ETO*-mutant AML cells (Figure 2). Nonetheless, PARPi appear to have limited activity as single agents in treatment of relapsed or refractory AML cells. Therefore, combination therapy has emerged and is anticipated to expand the efficacy of PARPi against AML. Indeed, several studies have tested the efficacy of combination therapy with PARPi and different small molecules, such as antibody-drug conjugates, FLT3 inhibitor, DNMTi, and HDACi, in AML cells.

As HR-related genes are rarely mutated in AML, the therapeutic efficacy of PARP inhibitors is limited in this disease. Expanding the utility of PARP inhibitor is an urgent need in the clinic and will satisfy more AML patients. ‘BRCAness’ is termed as a subset of tumors that lack *BRCA* mutations but show other characteristics that can phenocopy *BRCA* mutation. Owing to competent HR activity in AML cells, inducing ‘BRCAness’ phenotype might have synthetic lethality effects with PARP inhibitors in treating AML. Indeed, various studies have identified strategies that induce ‘BRCAness’ to treat AML. A newest study indicated that targeting splicing factor SF3B1 resulted in impaired DNA damage response and sensitized AML cells to PARP inhibitor (Wheeler et al., 2024). Epigenetic dysregulation contributes to AML pathogenesis. Furthermore, targeting KDM6 demethylase sensitizes AML cells to PARP inhibitor. Besides, targeting epigenetic factors such as BRD4 have shown synergistic antitumor effects with PARP inhibitor in a variety of cancer treatment, such combination therapy needs further exploration in the treatment AML (Yang et al., 2017; Sun et al., 2018). Together, future directions, both computational or experimental methodologies, should focus on identifying cancer-specific synthetic lethality interactions to extent the treatment efficacy of PARP inhibitor in AML.

Over the past decade, identifying suitable immune therapeutics to suppress leukemic cells and their progenitor cells have been a focus of cancer research. Unfortunately, the immunosuppressive microenvironment of acute leukemia supports leukemic cell evasion of immune cell attack. Therefore, considering the complexity of the AML tumor microenvironment, a rational combination of immunotherapy with complementary strategies can be predicted to prevent tumor escape and eradicate AML cells, without increased side effects. In this context, understanding the key role of the tumor microenvironment in hampering therapeutic efficacy and modulating toxicity warrants greater attention.

Future challenges for AML therapeutics include increasing treatment efficacy and regulating immune escape mechanisms generated by the tumor microenvironment. Furthermore, precise identification of the mechanisms of immune evasion in individual patients with AML has potential to inform development of personalized immunotherapy, according to specific immune signatures. Since the combination of an immune checkpoint inhibitor and PARPi induce robust antitumor immune responses, particularly against cancers with high levels of HR deficiency, investigating novel immune therapy combinations may be beneficial to more patients with acute leukemia, regardless of their genetic background. Further, evaluation of treatment efficacy and safety in preclinical and clinical studies, and identifying predictive biomarkers for patient selection, should be priorities.

## References

[B1] Abbotts R. Wilson D. M. 3rd (2017). Coordination of DNA single strand break repair. Free Radic. Biol. Med. 107, 228–244. 10.1016/j.freeradbiomed.2016.11.039 27890643 PMC5443707

[B2] Al-Matary Y. S. Botezatu L. Opalka B. Hones J. M. Lams R. F. Thivakaran A. (2016). Acute myeloid leukemia cells polarize macrophages towards a leukemia supporting state in a Growth factor independence 1 dependent manner. Haematologica 101, 1216–1227. 10.3324/haematol.2016.143180 27390361 PMC5046651

[B3] Altmeyer M. Messner S. Hassa P. O. Fey M. Hottiger M. O. (2009). Molecular mechanism of poly(ADP-ribosyl)ation by PARP1 and identification of lysine residues as ADP-ribose acceptor sites. Nucleic Acids Res. 37, 3723–3738. 10.1093/nar/gkp229 19372272 PMC2699514

[B4] Ame J. C. Spenlehauer C. de Murcia G. (2004). The PARP superfamily. Bioessays 26, 882–893. 10.1002/bies.20085 15273990

[B5] Anand R. Ranjha L. Cannavo E. Cejka P. (2016). Phosphorylated CtIP functions as a Co-factor of the MRE11-RAD50-NBS1 endonuclease in DNA end resection. Mol. Cell. 64, 940–950. 10.1016/j.molcel.2016.10.017 27889449

[B6] Aurelius J. Thoren F. B. Akhiani A. A. Brune M. Palmqvist L. Hansson M. (2012). Monocytic AML cells inactivate antileukemic lymphocytes: role of NADPH oxidase/gp91(phox) expression and the PARP-1/PAR pathway of apoptosis. Blood 119, 5832–5837. 10.1182/blood-2011-11-391722 22550344 PMC3418695

[B7] Baer M. R. Kogan A. A. Bentzen S. M. Mi T. Lapidus R. G. Duong V. H. (2022). Phase I clinical trial of DNA methyltransferase inhibitor decitabine and PARP inhibitor talazoparib combination therapy in relapsed/refractory acute myeloid leukemia. Clin. Cancer Res. 28, 1313–1322. 10.1158/1078-0432.CCR-21-3729 35091444 PMC8976746

[B8] Boila L. D. Ghosh S. Bandyopadhyay S. K. Jin L. Murison A. Zeng A. G. X. (2023). KDM6 demethylases integrate DNA repair gene regulation and loss of KDM6A sensitizes human acute myeloid leukemia to PARP and BCL2 inhibition. Leukemia 37, 751–764. 10.1038/s41375-023-01833-z 36720973

[B9] Brabson J. P. Leesang T. Yap Y. S. Wang J. Lam M. Q. Fang B. (2023). Oxidized mC modulates synthetic lethality to PARP inhibitors for the treatment of leukemia. Cell. Rep. 42, 112027. 10.1016/j.celrep.2023.112027 36848231 PMC9989506

[B10] Bryant H. E. Petermann E. Schultz N. Jemth A. S. Loseva O. Issaeva N. (2009). PARP is activated at stalled forks to mediate Mre11-dependent replication restart and recombination. EMBO J. 28, 2601–2615. 10.1038/emboj.2009.206 19629035 PMC2738702

[B11] Bryant H. E. Schultz N. Thomas H. D. Parker K. M. Flower D. Lopez E. (2005). Specific killing of BRCA2-deficient tumours with inhibitors of poly(ADP-ribose) polymerase. Nature 434, 913–917. 10.1038/nature03443 15829966

[B12] Bunting S. F. Callen E. Wong N. Chen H. T. Polato F. Gunn A. (2010). 53BP1 inhibits homologous recombination in Brca1-deficient cells by blocking resection of DNA breaks. Cell. 141, 243–254. 10.1016/j.cell.2010.03.012 20362325 PMC2857570

[B13] Caldecott K. W. (2008). Single-strand break repair and genetic disease. Nat. Rev. Genet. 9, 619–631. 10.1038/nrg2380 18626472

[B14] Carreira S. Porta N. Arce-Gallego S. Seed G. Llop-Guevara A. Bianchini D. (2021). Biomarkers associating with PARP inhibitor benefit in prostate cancer in the TOPARP-B trial. Cancer Discov. 11, 2812–2827. 10.1158/2159-8290.CD-21-0007 34045297 PMC9414325

[B15] Casorelli I. Tenedini E. Tagliafico E. Blasi M. F. Giuliani A. Crescenzi M. (2006). Identification of a molecular signature for leukemic promyelocytes and their normal counterparts: focus on DNA repair genes. Leukemia 20, 1978–1988. 10.1038/sj.leu.2404376 16990782

[B16] Chang H. H. Y. Pannunzio N. R. Adachi N. Lieber M. R. (2017). Non-homologous DNA end joining and alternative pathways to double-strand break repair. Nat. Rev. Mol. Cell. Biol. 18, 495–506. 10.1038/nrm.2017.48 28512351 PMC7062608

[B17] Chapman J. R. Taylor M. R. Boulton S. J. (2012). Playing the end game: DNA double-strand break repair pathway choice. Mol. Cell. 47, 497–510. 10.1016/j.molcel.2012.07.029 22920291

[B18] Chen H. Lisby M. Symington L. S. (2013). RPA coordinates DNA end resection and prevents formation of DNA hairpins. Mol. Cell. 50, 589–600. 10.1016/j.molcel.2013.04.032 23706822 PMC3855855

[B19] Chen J. K. Lin W. L. Chen Z. Liu H. W. (2018). PARP-1-dependent recruitment of cold-inducible RNA-binding protein promotes double-strand break repair and genome stability. Proc. Natl. Acad. Sci. U. S. A. 115, E1759–E1768. 10.1073/pnas.1713912115 29432179 PMC5828585

[B20] Christopher M. J. Petti A. A. Rettig M. P. Miller C. A. Chendamarai E. Duncavage E. J. (2018). Immune escape of relapsed AML cells after allogeneic transplantation. N. Engl. J. Med. 379, 2330–2341. 10.1056/NEJMoa1808777 30380364 PMC6322675

[B21] Cruz-Garcia A. Lopez-Saavedra A. Huertas P. (2014). BRCA1 accelerates CtIP-mediated DNA-end resection. Cell. Rep. 9, 451–459. 10.1016/j.celrep.2014.08.076 25310973

[B22] Curti A. Trabanelli S. Salvestrini V. Baccarani M. Lemoli R. M. (2009). The role of indoleamine 2,3-dioxygenase in the induction of immune tolerance: focus on hematology. Blood 113, 2394–2401. 10.1182/blood-2008-07-144485 19023117

[B23] Curtin N. J. Szabo C. (2020). Poly(ADP-ribose) polymerase inhibition: past, present and future. Nat. Rev. Drug Discov. 19, 711–736. 10.1038/s41573-020-0076-6 32884152

[B24] Dall’Olio F. G. Marabelle A. Caramella C. Garcia C. Aldea M. Chaput N. (2022). Tumour burden and efficacy of immune-checkpoint inhibitors. Nat. Rev. Clin. Oncol. 19, 75–90. 10.1038/s41571-021-00564-3 34642484

[B25] Daver N. Garcia-Manero G. Basu S. Boddu P. C. Alfayez M. Cortes J. E. (2019). Efficacy, safety, and biomarkers of response to azacitidine and nivolumab in relapsed/refractory acute myeloid leukemia: a nonrandomized, open-label, phase II study. Cancer Discov. 9, 370–383. 10.1158/2159-8290.CD-18-0774 30409776 PMC6397669

[B26] Davids M. S. Kim H. T. Bachireddy P. Costello C. Liguori R. Savell A. (2016). Ipilimumab for patients with relapse after allogeneic transplantation. N. Engl. J. Med. 375, 143–153. 10.1056/NEJMoa1601202 27410923 PMC5149459

[B27] Davis A. J. Chen D. J. (2013). DNA double strand break repair via non-homologous end-joining. Transl. Cancer Res. 2, 130–143. 10.3978/j.issn.2218-676X.2013.04.02 24000320 PMC3758668

[B28] Demin A. A. Hirota K. Tsuda M. Adamowicz M. Hailstone R. Brazina J. (2021). XRCC1 prevents toxic PARP1 trapping during DNA base excision repair. Mol. Cell. 81, 3018–3030.e5. 10.1016/j.molcel.2021.05.009 34102106 PMC8294329

[B29] Dillon K. M. Bekele R. T. Sztupinszki Z. Hanlon T. Rafiei S. Szallasi Z. (2022). PALB2 or BARD1 loss confers homologous recombination deficiency and PARP inhibitor sensitivity in prostate cancer. NPJ Precis. Oncol. 6, 49. 10.1038/s41698-022-00291-7 35768576 PMC9242979

[B30] Ding L. Kim H. J. Wang Q. Kearns M. Jiang T. Ohlson C. E. (2018). PARP inhibition elicits STING-dependent antitumor immunity in brca1-deficient ovarian cancer. Cell. Rep. 25, 2972–2980. 10.1016/j.celrep.2018.11.054 30540933 PMC6366450

[B31] El-Khamisy S. F. Masutani M. Suzuki H. Caldecott K. W. (2003). A requirement for PARP-1 for the assembly or stability of XRCC1 nuclear foci at sites of oxidative DNA damage. Nucleic Acids Res. 31, 5526–5533. 10.1093/nar/gkg761 14500814 PMC206461

[B32] Escribano-Diaz C. Orthwein A. Fradet-Turcotte A. Xing M. Young J. T. Tkac J. (2013). A cell cycle-dependent regulatory circuit composed of 53BP1-RIF1 and BRCA1-CtIP controls DNA repair pathway choice. Mol. Cell. 49, 872–883. 10.1016/j.molcel.2013.01.001 23333306

[B33] Esposito M. T. Zhao L. Fung T. K. Rane J. K. Wilson A. Martin N. (2015). Synthetic lethal targeting of oncogenic transcription factors in acute leukemia by PARP inhibitors. Nat. Med. 21, 1481–1490. 10.1038/nm.3993 26594843

[B34] Estey E. H. (2014). Acute myeloid leukemia: 2014 update on risk-stratification and management. Am. J. Hematol. 89, 1063–1081. 10.1002/ajh.23834 25318680

[B35] Falzacappa M. V. Ronchini C. Faretta M. Iacobucci I. Di Rora A. G. Martinelli G. (2015). The combination of the PARP inhibitor rucaparib and 5FU is an effective strategy for treating acute leukemias. Mol. Cancer Ther. 14, 889–898. 10.1158/1535-7163.MCT-14-0276 25667168

[B36] Faraoni I. Aloisio F. De Gabrieli A. Consalvo M. I. Lavorgna S. Voso M. T. (2018). The poly(ADP-ribose) polymerase inhibitor olaparib induces up-regulation of death receptors in primary acute myeloid leukemia blasts by NF-κB activation. Cancer Lett. 423, 127–138. 10.1016/j.canlet.2018.03.008 29526802

[B37] Farmer H. McCabe N. Lord C. J. Tutt A. N. Johnson D. A. Richardson T. B. (2005). Targeting the DNA repair defect in BRCA mutant cells as a therapeutic strategy. Nature 434, 917–921. 10.1038/nature03445 15829967

[B38] Fritz C. Portwood S. M. Przespolewski A. Wang E. S. (2021). PARP goes the weasel! Emerging role of PARP inhibitors in acute leukemias. Blood Rev. 45, 100696. 10.1016/j.blre.2020.100696 32482307 PMC9295907

[B39] Garcia T. B. Snedeker J. C. Baturin D. Gardner L. Fosmire S. P. Zhou C. (2017). A small-molecule inhibitor of WEE1, AZD1775, synergizes with olaparib by impairing homologous recombination and enhancing DNA damage and apoptosis in acute leukemia. Mol. Cancer Ther. 16, 2058–2068. 10.1158/1535-7163.MCT-16-0660 28655785 PMC5628125

[B40] Gasser S. Orsulic S. Brown E. J. Raulet D. H. (2005). The DNA damage pathway regulates innate immune system ligands of the NKG2D receptor. Nature 436, 1186–1190. 10.1038/nature03884 15995699 PMC1352168

[B41] Gbyli R. Song Y. Liu W. Gao Y. Biancon G. Chandhok N. S. (2022). *In vivo* anti-tumor effect of PARP inhibition in IDH1/2 mutant MDS/AML resistant to targeted inhibitors of mutant IDH1/2. Leukemia 36, 1313–1323. 10.1038/s41375-022-01536-x 35273342 PMC9103411

[B42] Goswami M. Gui G. Dillon L. W. Lindblad K. E. Thompson J. Valdez J. (2022). Pembrolizumab and decitabine for refractory or relapsed acute myeloid leukemia. J. Immunother. Cancer 10, e003392. 10.1136/jitc-2021-003392 35017151 PMC8753450

[B43] Grellety T. Peyraud F. Sevenet N. Tredan O. Dohollou N. Barouk-Simonet E. (2020). Dramatic response to PARP inhibition in a PALB2-mutated breast cancer: moving beyond BRCA. Ann. Oncol. 31, 822–823. 10.1016/j.annonc.2020.03.283 32194151

[B44] Han Y. Jin F. Xie Y. Liu Y. Hu S. Liu X. D. (2019). DNAPKcs PARylation regulates DNAPK kinase activity in the DNA damage response. Mol. Med. Rep. 20, 3609–3616. 10.3892/mmr.2019.10640 31485633 PMC6755157

[B45] Hansrivijit P. Gale R. P. Barrett J. Ciurea S. O. (2019). Cellular therapy for acute myeloid Leukemia - current status and future prospects. Blood Rev. 37, 100578. 10.1016/j.blre.2019.05.002 31109711

[B46] Hanzlikova H. Gittens W. Krejcikova K. Zeng Z. Caldecott K. W. (2017). Overlapping roles for PARP1 and PARP2 in the recruitment of endogenous XRCC1 and PNKP into oxidized chromatin. Nucleic Acids Res. 45, 2546–2557. 10.1093/nar/gkw1246 27965414 PMC5389470

[B47] Hartlerode A. J. Scully R. (2009). Mechanisms of double-strand break repair in somatic mammalian cells. Biochem. J. 423, 157–168. 10.1042/BJ20090942 19772495 PMC2983087

[B48] Hewitt G. Borel V. Segura-Bayona S. Takaki T. Ruis P. Bellelli R. (2021). Defective ALC1 nucleosome remodeling confers PARPi sensitization and synthetic lethality with HRD. Mol. Cell. 81, 767–783.e11. 10.1016/j.molcel.2020.12.006 33333017 PMC7895907

[B49] Huertas P. Cortes-Ledesma F. Sartori A. A. Aguilera A. Jackson S. P. (2008). CDK targets Sae2 to control DNA-end resection and homologous recombination. Nature 455, 689–692. 10.1038/nature07215 18716619 PMC2635538

[B50] Jeppsson K. Kanno T. Shirahige K. Sjogren C. (2014). The maintenance of chromosome structure: positioning and functioning of SMC complexes. Nat. Rev. Mol. Cell. Biol. 15, 601–614. 10.1038/nrm3857 25145851

[B51] Ji Y. Tulin A. V. (2010). The roles of PARP1 in gene control and cell differentiation. Curr. Opin. Genet. Dev. 20, 512–518. 10.1016/j.gde.2010.06.001 20591646 PMC2942995

[B52] Jubin T. Kadam A. Jariwala M. Bhatt S. Sutariya S. Gani A. R. (2016). The PARP family: insights into functional aspects of poly (ADP-ribose) polymerase-1 in cell growth and survival. Cell. Prolif. 49, 421–437. 10.1111/cpr.12268 27329285 PMC6496725

[B53] Juhasz S. Elbakry A. Mathes A. Lobrich M. (2018). ATRX promotes DNA repair synthesis and sister chromatid exchange during homologous recombination. Mol. Cell. 71, 11–24. 10.1016/j.molcel.2018.05.014 29937341

[B54] Kausar T. Schreiber J. S. Karnak D. Parsels L. A. Parsels J. D. Davis M. A. (2015). Sensitization of pancreatic cancers to gemcitabine chemoradiation by WEE1 kinase inhibition depends on homologous recombination repair. Neoplasia 17, 757–766. 10.1016/j.neo.2015.09.006 26585231 PMC4656803

[B55] Kent T. Chandramouly G. McDevitt S. M. Ozdemir A. Y. Pomerantz R. T. (2015). Mechanism of microhomology-mediated end-joining promoted by human DNA polymerase θ. Nat. Struct. Mol. Biol. 22, 230–237. 10.1038/nsmb.2961 25643323 PMC4351179

[B56] Kikushige Y. Miyamoto T. (2015). Identification of TIM-3 as a leukemic stem cell surface molecule in primary acute myeloid leukemia. Oncology 89 (Suppl. 1), 28–32. 10.1159/000431062 26551150

[B57] Kikushige Y. Shima T. Takayanagi S. Urata S. Miyamoto T. Iwasaki H. (2010). TIM-3 is a promising target to selectively kill acute myeloid leukemia stem cells. Cell. Stem Cell. 7, 708–717. 10.1016/j.stem.2010.11.014 21112565

[B58] Kim C. Wang X. D. Yu Y. (2020). PARP1 inhibitors trigger innate immunity via PARP1 trapping-induced DNA damage response. Elife 9, e60637. 10.7554/eLife.60637 32844745 PMC7486119

[B59] Kohl V. Flach J. Naumann N. Brendel S. Kleiner H. Weiss C. (2019). Antileukemic efficacy *in vitro* of talazoparib and APE1 inhibitor III combined with decitabine in myeloid malignancies. Cancers (Basel) 11, 1493. 10.3390/cancers11101493 31623402 PMC6826540

[B60] Kornepati A. V. R. Boyd J. T. Murray C. E. Saifetiarova J. de la Pena Avalos B. Rogers C. M. (2022). Tumor intrinsic PD-L1 promotes DNA repair in distinct cancers and suppresses PARP inhibitor-induced synthetic lethality. Cancer Res. 82, 2156–2170. 10.1158/0008-5472.CAN-21-2076 35247877 PMC9987177

[B61] Krishnakumar R. Kraus W. L. (2010). PARP-1 regulates chromatin structure and transcription through a KDM5B-dependent pathway. Mol. Cell. 39, 736–749. 10.1016/j.molcel.2010.08.014 20832725 PMC2939044

[B62] Langelier M. F. Planck J. L. Roy S. Pascal J. M. (2011). Crystal structures of poly(ADP-ribose) polymerase-1 (PARP-1) zinc fingers bound to DNA: structural and functional insights into DNA-dependent PARP-1 activity. J. Biol. Chem. 286, 10690–10701. 10.1074/jbc.M110.202507 21233213 PMC3060520

[B63] Li D. Luo Y. Chen X. Zhang L. Wang T. Zhuang Y. (2019). NF-κB and poly (ADP-ribose) polymerase 1 form a positive feedback loop that regulates DNA repair in acute myeloid leukemia cells. Mol. Cancer Res. 17, 761–772. 10.1158/1541-7786.MCR-18-0523 30559256

[B64] Li M. Yu X. (2013). Function of BRCA1 in the DNA damage response is mediated by ADP-ribosylation. Cancer Cell. 23, 693–704. 10.1016/j.ccr.2013.03.025 23680151 PMC3759356

[B65] Li X. Li C. Jin J. Wang J. Huang J. Ma Z. (2018). High PARP-1 expression predicts poor survival in acute myeloid leukemia and PARP-1 inhibitor and SAHA-bendamustine hybrid inhibitor combination treatment synergistically enhances anti-tumor effects. EBioMedicine 38, 47–56. 10.1016/j.ebiom.2018.11.025 30472087 PMC6306376

[B66] Lieber M. R. (2010). The mechanism of double-strand DNA break repair by the nonhomologous DNA end-joining pathway. Annu. Rev. Biochem. 79, 181–211. 10.1146/annurev.biochem.052308.093131 20192759 PMC3079308

[B67] Liu T. Huang J. (2014). Quality control of homologous recombination. Cell. Mol. Life Sci. 71, 3779–3797. 10.1007/s00018-014-1649-5 24858417 PMC11114062

[B68] Lu T. Gabrilovich D. I. (2012). Molecular pathways: tumor-infiltrating myeloid cells and reactive oxygen species in regulation of tumor microenvironment. Clin. Cancer Res. 18, 4877–4882. 10.1158/1078-0432.CCR-11-2939 22718858 PMC3445728

[B69] Luedeman M. E. Stroik S. Feng W. Luthman A. J. Gupta G. P. Ramsden D. A. (2022). Poly(ADP) ribose polymerase promotes DNA polymerase theta-mediated end joining by activation of end resection. Nat. Commun. 13, 4547. 10.1038/s41467-022-32166-7 35927262 PMC9352658

[B70] Maifrede S. Nieborowska-Skorska M. Sullivan-Reed K. Dasgupta Y. Podszywalow-Bartnicka P. Le B. V. (2018). Tyrosine kinase inhibitor-induced defects in DNA repair sensitize FLT3(ITD)-positive leukemia cells to PARP1 inhibitors. Blood 132, 67–77. 10.1182/blood-2018-02-834895 29784639 PMC6034642

[B71] Meng X. W. Koh B. D. Zhang J. S. Flatten K. S. Schneider P. A. Billadeau D. D. (2014). Poly(ADP-ribose) polymerase inhibitors sensitize cancer cells to death receptor-mediated apoptosis by enhancing death receptor expression. J. Biol. Chem. 289, 20543–20558. 10.1074/jbc.M114.549220 24895135 PMC4110268

[B72] Moison C. Chagraoui J. Caron M. C. Gagne J. P. Coulombe Y. Poirier G. G. (2021). Zinc finger protein E4F1 cooperates with PARP-1 and BRG1 to promote DNA double-strand break repair. Proc. Natl. Acad. Sci. U. S. A. 118, e2019408118. 10.1073/pnas.2019408118 33692124 PMC7980444

[B73] Molenaar R. J. Radivoyevitch T. Nagata Y. Khurshed M. Przychodzen B. Makishima H. (2018). IDH1/2 mutations sensitize acute myeloid leukemia to PARP inhibition and this is reversed by IDH1/2-mutant inhibitors. Clin. Cancer Res. 24, 1705–1715. 10.1158/1078-0432.CCR-17-2796 29339439 PMC5884732

[B74] Murai J. Huang S. Y. Das B. B. Renaud A. Zhang Y. Doroshow J. H. (2012). Trapping of PARP1 and PARP2 by clinical PARP inhibitors. Cancer Res. 72, 5588–5599. 10.1158/0008-5472.CAN-12-2753 23118055 PMC3528345

[B75] Muvarak N. E. Chowdhury K. Xia L. Robert C. Choi E. Y. Cai Y. (2016). Enhancing the cytotoxic effects of PARP inhibitors with DNA demethylating agents - a potential therapy for cancer. Cancer Cell. 30, 637–650. 10.1016/j.ccell.2016.09.002 27728808 PMC5201166

[B76] Okano S. Lan L. Tomkinson A. E. Yasui A. (2005). Translocation of XRCC1 and DNA ligase IIIalpha from centrosomes to chromosomes in response to DNA damage in mitotic human cells. Nucleic Acids Res. 33, 422–429. 10.1093/nar/gki190 15653642 PMC546168

[B77] Orta M. L. Hoglund A. Calderon-Montano J. M. Dominguez I. Burgos-Moron E. Visnes T. (2014). The PARP inhibitor Olaparib disrupts base excision repair of 5-aza-2'-deoxycytidine lesions. Nucleic Acids Res. 42, 9108–9120. 10.1093/nar/gku638 25074383 PMC4132747

[B78] Orthwein A. Fradet-Turcotte A. Noordermeer S. M. Canny M. D. Brun C. M. Strecker J. (2014). Mitosis inhibits DNA double-strand break repair to guard against telomere fusions. Science 344, 189–193. 10.1126/science.1248024 24652939

[B79] Paczulla A. M. Rothfelder K. Raffel S. Konantz M. Steinbacher J. Wang H. (2019). Absence of NKG2D ligands defines leukaemia stem cells and mediates their immune evasion. Nature 572, 254–259. 10.1038/s41586-019-1410-1 31316209 PMC6934414

[B80] Paes Dias M. Tripathi V. van der Heijden I. Cong K. Manolika E. M. Bhin J. (2021). Loss of nuclear DNA ligase III reverts PARP inhibitor resistance in BRCA1/53BP1 double-deficient cells by exposing ssDNA gaps. Mol. Cell. 81, 4692–4708.e9. 10.1016/j.molcel.2021.09.005 34555355 PMC9098260

[B81] Pan J. Wang Y. Huang S. Mao S. Ling Q. Li C. (2024). High expression of BCAT1 sensitizes AML cells to PARP inhibitor by suppressing DNA damage response. J. Mol. Med. Berl. 102, 415–433. 10.1007/s00109-023-02409-1 38340163

[B82] Pardo B. Gomez-Gonzalez B. Aguilera A. (2009). DNA repair in mammalian cells: DNA double-strand break repair: how to fix a broken relationship. Cell. Mol. Life Sci. 66, 1039–1056. 10.1007/s00018-009-8740-3 19153654 PMC11131446

[B83] Parvin S. Ramirez-Labrada A. Aumann S. Lu X. Weich N. Santiago G. (2019). LMO2 confers synthetic lethality to PARP inhibition in DLBCL. Cancer Cell. 36, 237–249. 10.1016/j.ccell.2019.07.007 31447348 PMC6752209

[B84] Pham M. M. Ngoi N. Y. L. Peng G. Tan D. S. P. Yap T. A. (2021). Development of poly(ADP-ribose) polymerase inhibitor and immunotherapy combinations: progress, pitfalls, and promises. Trends Cancer 7, 958–970. 10.1016/j.trecan.2021.05.004 34158277 PMC8458234

[B85] Prakash R. Zhang Y. Feng W. Jasin M. (2015). Homologous recombination and human health: the roles of BRCA1, BRCA2, and associated proteins. Cold Spring Harb. Perspect. Biol. 7, a016600. 10.1101/cshperspect.a016600 25833843 PMC4382744

[B86] Pyzer A. R. Stroopinsky D. Rajabi H. Washington A. Tagde A. Coll M. (2017). MUC1-mediated induction of myeloid-derived suppressor cells in patients with acute myeloid leukemia. Blood 129, 1791–1801. 10.1182/blood-2016-07-730614 28126925 PMC5813734

[B87] Ranjha L. Howard S. M. Cejka P. (2018). Main steps in DNA double-strand break repair: an introduction to homologous recombination and related processes. Chromosoma 127, 187–214. 10.1007/s00412-017-0658-1 29327130

[B88] Ronson G. E. Piberger A. L. Higgs M. R. Olsen A. L. Stewart G. S. McHugh P. J. (2018). PARP1 and PARP2 stabilise replication forks at base excision repair intermediates through Fbh1-dependent Rad51 regulation. Nat. Commun. 9, 746. 10.1038/s41467-018-03159-2 29467415 PMC5821833

[B89] San Filippo J. Sung P. Klein H. (2008). Mechanism of eukaryotic homologous recombination. Annu. Rev. Biochem. 77, 229–257. 10.1146/annurev.biochem.77.061306.125255 18275380

[B90] Schiewer M. J. Goodwin J. F. Han S. Brenner J. C. Augello M. A. Dean J. L. (2012). Dual roles of PARP-1 promote cancer growth and progression. Cancer Discov. 2, 1134–1149. 10.1158/2159-8290.CD-12-0120 22993403 PMC3519969

[B91] Schreiber V. Dantzer F. Ame J. C. de Murcia G. (2006). Poly(ADP-ribose): novel functions for an old molecule. Nat. Rev. Mol. Cell. Biol. 7, 517–528. 10.1038/nrm1963 16829982

[B92] Sen T. Rodriguez B. L. Chen L. Corte C. M. D. Morikawa N. Fujimoto J. (2019). Targeting DNA damage response promotes antitumor immunity through STING-mediated T-cell activation in small cell lung cancer. Cancer Discov. 9, 646–661. 10.1158/2159-8290.CD-18-1020 30777870 PMC6563834

[B93] Shen Y. Aoyagi-Scharber M. Wang B. (2015). Trapping poly(ADP-ribose) polymerase. J. Pharmacol. Exp. Ther. 353, 446–457. 10.1124/jpet.114.222448 25758918

[B94] Slade D. (2019). Mitotic functions of poly(ADP-ribose) polymerases. Biochem. Pharmacol. 167, 33–43. 10.1016/j.bcp.2019.03.028 30910692 PMC7056360

[B95] Stavropoulou V. Kaspar S. Brault L. Sanders M. A. Juge S. Morettini S. (2016). MLL-AF9 expression in hematopoietic stem cells drives a highly invasive AML expressing EMT-related genes linked to poor outcome. Cancer Cell. 30, 43–58. 10.1016/j.ccell.2016.05.011 27344946

[B96] Sulkowski P. L. Corso C. D. Robinson N. D. Scanlon S. E. Purshouse K. R. Bai H. (2017). 2-Hydroxyglutarate produced by neomorphic IDH mutations suppresses homologous recombination and induces PARP inhibitor sensitivity. Sci. Transl. Med. 9, eaal2463. 10.1126/scitranslmed.aal2463 28148839 PMC5435119

[B97] Sun C. Yin J. Fang Y. Chen J. Jeong K. J. Chen X. (2018). BRD4 inhibition is synthetic lethal with PARP inhibitors through the induction of homologous recombination deficiency. Cancer Cell. 33, 401–416. 10.1016/j.ccell.2018.01.019 29533782 PMC5944839

[B98] Sun H. Li Y. Zhang Z. F. Ju Y. Li L. Zhang B. C. (2015). Increase in myeloid-derived suppressor cells (MDSCs) associated with minimal residual disease (MRD) detection in adult acute myeloid leukemia. Int. J. Hematol. 102, 579–586. 10.1007/s12185-015-1865-2 26358057

[B99] Syed Y. Y. (2017). Rucaparib: first global approval. Drugs 77, 585–592. 10.1007/s40265-017-0716-2 28247266

[B100] Taghiloo S. Asgarian-Omran H. (2021). Immune evasion mechanisms in acute myeloid leukemia: a focus on immune checkpoint pathways. Crit. Rev. Oncol. Hematol. 157, 103164. 10.1016/j.critrevonc.2020.103164 33271388

[B101] Tettamanti S. Pievani A. Biondi A. Dotti G. Serafini M. (2022). Catch me if you can: how AML and its niche escape immunotherapy. Leukemia 36, 13–22. 10.1038/s41375-021-01350-x 34302116 PMC8727297

[B102] Tothova Z. Valton A. L. Gorelov R. A. Vallurupalli M. Krill-Burger J. M. Holmes A. (2021). Cohesin mutations alter DNA damage repair and chromatin structure and create therapeutic vulnerabilities in MDS/AML. JCI Insight 6, e142149. 10.1172/jci.insight.142149 33351783 PMC7934867

[B103] Tyner J. W. Tognon C. E. Bottomly D. Wilmot B. Kurtz S. E. Savage S. L. (2018). Functional genomic landscape of acute myeloid leukaemia. Nature 562, 526–531. 10.1038/s41586-018-0623-z 30333627 PMC6280667

[B104] Vago L. Gojo I. (2020). Immune escape and immunotherapy of acute myeloid leukemia. J. Clin. Investig. 130, 1552–1564. 10.1172/JCI129204 32235097 PMC7108895

[B105] Valdez B. C. Li Y. Murray D. Liu Y. Nieto Y. Champlin R. E. (2018). Combination of a hypomethylating agent and inhibitors of PARP and HDAC traps PARP1 and DNMT1 to chromatin, acetylates DNA repair proteins, down-regulates NuRD and induces apoptosis in human leukemia and lymphoma cells. Oncotarget 9, 3908–3921. 10.18632/oncotarget.23386 29423093 PMC5790510

[B106] Wacker D. A. Ruhl D. D. Balagamwala E. H. Hope K. M. Zhang T. Kraus W. L. (2007). The DNA binding and catalytic domains of poly(ADP-ribose) polymerase 1 cooperate in the regulation of chromatin structure and transcription. Mol. Cell. Biol. 27, 7475–7485. 10.1128/MCB.01314-07 17785446 PMC2169059

[B107] Wheeler E. C. Martin B. J. E. Doyle W. C. Neaher S. Conway C. A. Pitton C. N. (2024). Splicing modulators impair DNA damage response and induce killing of cohesin-mutant MDS and AML. Sci. Transl. Med. 16, eade2774. 10.1126/scitranslmed.ade2774 38170787 PMC11222919

[B108] Wu J. Huen M. S. Lu L. Y. Ye L. Dou Y. Ljungman M. (2009). Histone ubiquitination associates with BRCA1-dependent DNA damage response. Mol. Cell. Biol. 29, 849–860. 10.1128/MCB.01302-08 19015238 PMC2630672

[B109] Wyatt D. W. Feng W. Conlin M. P. Yousefzadeh M. J. Roberts S. A. Mieczkowski P. (2016). Essential roles for polymerase theta-mediated end joining in the repair of chromosome breaks. Mol. Cell. 63, 662–673. 10.1016/j.molcel.2016.06.020 27453047 PMC4992412

[B110] Xie A. Kwok A. Scully R. (2009). Role of mammalian Mre11 in classical and alternative nonhomologous end joining. Nat. Struct. Mol. Biol. 16, 814–818. 10.1038/nsmb.1640 19633669 PMC2730592

[B111] Yang L. Zhang Y. Shan W. Hu Z. Yuan J. Pi J. (2017). Repression of BET activity sensitizes homologous recombination-proficient cancers to PARP inhibition. Sci. Transl. Med. 9, eaal1645. 10.1126/scitranslmed.aal1645 28747513 PMC5705017

[B112] Yang Y. Li C. Liu T. Dai X. Bazhin A. V. (2020). Myeloid-derived suppressor cells in tumors: from mechanisms to antigen specificity and microenvironmental regulation. Front. Immunol. 11, 1371. 10.3389/fimmu.2020.01371 32793192 PMC7387650

[B113] Yu W. Lescale C. Babin L. Bedora-Faure M. Lenden-Hasse H. Baron L. (2020). Repair of G1 induced DNA double-strand breaks in S-G2/M by alternative NHEJ. Nat. Commun. 11, 5239. 10.1038/s41467-020-19060-w 33067475 PMC7567796

[B114] Zeiser R. Vago L. (2019). Mechanisms of immune escape after allogeneic hematopoietic cell transplantation. Blood 133, 1290–1297. 10.1182/blood-2018-10-846824 30578254

[B115] Zhao F. Kim W. Kloeber J. A. Lou Z. (2020). DNA end resection and its role in DNA replication and DSB repair choice in mammalian cells. Exp. Mol. Med. 52, 1705–1714. 10.1038/s12276-020-00519-1 33122806 PMC8080561

